# Completeness and representativeness of small area socioeconomic data linked with the UK Clinical Practice Research Datalink (CPRD)

**DOI:** 10.1136/jech-2022-219200

**Published:** 2022-07-28

**Authors:** Preveina Mahadevan, Mia Harley, Stuart Fordyce, Susan Hodgson, Rebecca Ghosh, Puja Myles, Helen Booth, Eleanor Axson

**Affiliations:** CPRD, Medicines and Healthcare Products Regulatory Agency, London, UK

**Keywords:** epidemiology, public health, social class, biostatistics

## Abstract

**Background:**

The Clinical Practice Research Datalink (CPRD) holds primary care electronic healthcare records for 25% of the UK population. CPRD data can be linked via practice postcode in the UK, and additionally via patient postcode in England, to area-level socioeconomic status (SES) data including the Index of Multiple Deprivation (IMD), the Carstairs Index and the Townsend Deprivation Index; as well as rural–urban classification (RUC). This study aims to describe the completeness and representativeness of CPRD-linked SES and RUC data.

**Methods:**

Patients currently registered at general practices contributing data to the May 2021 snapshots of CPRD GOLD (n=445 587) and CPRD Aurum (n=13 278 825) were used to assess the completeness and representativeness of CPRD-linked SES and RUC data against the UK general population.

**Results:**

All currently registered patients had complete SES and RUC data at practice level across the UK. Most English patients in CPRD GOLD (78%), CPRD Aurum (94%) and combined (93%) had SES and RUC data at patient level. Patient-level SES data in CPRD for England were comparable to England’s general population (average IMD decile in CPRD 5.52±0.00 vs 5.50±0.02). CPRD UK practices were on average in more deprived areas than the UK general population (6.06±0.07 vs 5.50±0.02). A slightly higher proportion of CPRD patients and practices were from urban areas (85%) as compared with the UK general population (82%).

**Conclusion:**

Completeness of CPRD-linked SES and RUC data is high. The CPRD populations were broadly representative of the general populations in the UK in terms of SES and RUC.

**WHAT IS ALREADY KNOWN ON THIS TOPIC**

- Clinical Practice Research Datalink (CPRD)-linked area-level socioeconomic status (SES) measures estimate relative deprivation of a patient or general practice based on a variety of socioeconomic measures associated with their small geographical area. It is an essential health determinant indicator and useful for healthcare research. This study assesses the completeness and representation of CPRD-linked area-level SES measures compared with the UK general population.

**WHAT THIS STUDY ADDS**

- Overall, this study confirms that the completeness of the CPRD-linked area-level SES is high, CPRD practice-level SES and rural–urban classification (RUC) data is 100% complete across the UK and most English patients in CPRD GOLD (78%), CPRD Aurum (94%), and combined (93%) had patient-level SES and RUC data. CPRD patient population is broadly representative of the general population in England for patient-level SES, but CPRD practices are from slightly more deprived areas in the UK. The study supports researchers to make appropriate choices on use of small area data for public health research and benefit.

**HOW THIS STUDY MIGHT AFFECT RESEARCH, PRACTICE OR POLICY**

- This study provides advice for researchers using deprivation measures in combination with CPRD. Studies that use patient-level SES as a main exposure, researchers are advised to focus on patients in England from the combined CPRD databases, rather than using UK-wide data and substituting practice SES for patient data where this is absent. Studies wishing to use patient-level SES as a descriptor, stratifying variable and/or covariate should consider whether the practice-level measure is a sufficient proxy for their study design and note this in the methodology and/or limitations of any resulting output.

## Introduction

The Clinical Practice Research Datalink (CPRD) databases comprise a sample of primary care electronic healthcare records (EHRs) in the UK for over 62 million historical patients, of whom 16 million are currently registered patients. CPRD collects anonymised patient data from a network of general practices (GPs) across the UK. There are two CPRD primary care databases. CPRD GOLD consists of data sourced from GPs using Vision EHR software, whereas CPRD Aurum consists of data sourced from GPs using EMIS EHR software. CPRD data have been used for public health research for over 30 years, and more than 2900 peer-reviewed publications have used these data. Primary care data from CPRD are linked to a range of other datasets, including small area socioeconomic status (SES) and rural–urban classification (RUC) data to provide a fuller picture of health in the UK.

CPRD-linked area-level SES measures estimate relative deprivation of a patient or GP based on a variety of socioeconomic measures associated with their small geographical area. Different SES measures consider combinations of income, education, employment, access to public/health services, indoor/outdoor environments and proxies of these, such as owning a car. Studies have shown that SES measures are just as important as tobacco use, unhealthy diet, physical inactivity and harmful use of alcohol in predicting health outcomes.^1–3^ Area-level SES is not just a health determinant but is useful in healthcare research for applications such as planning and targeting of health and social care services and addressing and lowering health inequalities.

This study explored the following objectives: (1) the completeness of the CPRD-linked SES and RUC data; (2) the agreement and correlation between the different patient-level and practice-level CPRD-linked SES and RUC data in England and (3) the representativeness of CPRD-linked SES and RUC data against the general population in the UK, Great Britain (GB), and the individual nations.

The results will enable researchers to further understand the CPRD-linked SES data offerings by describing their completeness, usability, limitations and representativeness. This will help to inform choice of data sources, interpretation of results, and translation of those results into public health improvements for patients.

## Methods

The study population included all currently registered, acceptable patients through May 2021 builds of CPRD GOLD^4^ and CPRD Aurum.^5^ These were patients permanently registered at actively contributing practices, excluding transferred out and deceased patients. Over 80% of permanent registrations were deemed to be acceptable for use in research^6^ (online supplemental figure 1).

10.1136/jech-2022-219200.supp1 Supplementary data

**Figure 1:**
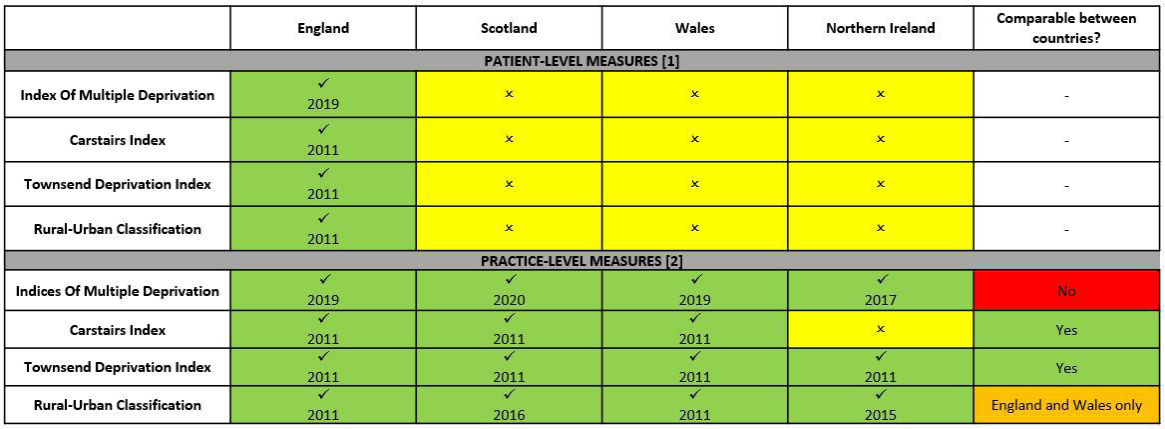
Availability of linked small area datasets in the Clinical Practice Research Datalink (CPRD) at the (top) patient level or (bottom) practice level, including the year of the dataset, and the comparability of measures between countries for each dataset. Linkages are available for both the CPRD GOLD and the CPRD Aurum databases.

Currently, CPRD data are linked to the following area-level SES data^7–10^: Indices of Multiple Deprivation (IMD) composite and domains, Carstairs Index, Townsend Deprivation Index; as well as RUC. The availabilities of the different CPRD-linked SES and RUC data are outlined in figure 1. As standard, researchers are provided only one patient level and/or one practice level CPRD-linked SES data for a single study, in order to prevent the possibility of deductive disclosure of the location of practices/patients.

CPRD-linked SES and RUC classifications are determined at a small area level associated with either a patient or a GP. All small areas in each UK nation are ranked according to their level of deprivation relative to that of other areas within that nation. The small area levels are: the lower layer super output area (SOA) geographical level in England and Wales (average of 1600 residents and a minimum of 300 households; the SOA level in Northern Ireland; and the data zone level in Scotland.^11^ The small areas are designed to be similar in population size and social characteristics, such as tenure of household and dwelling type. For approved studies, CPRD can provide linked SES or RUC measures for the small area associated with a patient (patient level) in England or with a GP (practice level) across the UK.

The IMD index is created by the UK Ministry of Housing, Communities and Local Government and uses mainly administrative data, such as benefit records, as well as census data. It provides both social domain-specific and composite scores based on weighted ranks for different social domains: income, education, employment, housing, environment, crime and access to services.^12–15^

The Townsend Deprivation Index covers all nations of the UK and is derived from weighted measures of unemployment, car ownership, house ownership and household overcrowding from the UK Census.^16^ The Carstairs Index covers all nations of GB and is also based on measures from the census.^17^ Unlike IMD, Townsend and Carstairs are comparable across nations of the UK (figure 1).

The RUC is a measure of urbanisation, based on resident population only, and does not reflect the land use, policy or financial characteristics of an area.^18–21^ The RUC is produced for England and Wales combined, but separate data sources and methodologies are used for Scotland and Northern Ireland making these non-comparable across nations.

Patient and practice small areas are assigned using the most recent postcode recorded for the patient or the practice. Patient postcodes are linked to their small area by National Health Service (NHS) Digital (NHSD), CPRD’s trusted third party, as CPRD does not hold identifiable patient information. Postcodes and small areas are linked using the NHS Postcode Directory (NHSPD).^22^ SES and RUC are then assigned to patients and practices based on their small area. Researchers using CPRD may then request the SES or RUC classifications at either the patient or practice level. Importantly, only the SES quantiles and RUC category are provided to researchers, the associated small area identifiers are not released.

The completeness of CPRD-linked SES and RUC data were assessed by calculating the count and proportion of patients with a given measure across the CPRD databases individually and combined. Agreement and correlation between different measures at patient level, and between patient and practice levels in England were assessed by calculating the count, Spearman correlation coefficients and proportion of patients with matching SES quintile rank at patient and practice levels. Quintiles were used as these are the most commonly requested quantile breakdown of SES ranks.

Representativeness of CPRD-linked SES and RUC at patient and practice level were compared with the most recently reported general population measures for the relevant UK nations.^12–15 18–20^ The mean±SD decile rank for each SES measure in each national population was defined as 5.50±0.02 because the definition of deciles requires that 50% of the national population place above the average and 50% place below the average. Deciles were used for a more detailed picture of representation.

## Results

### Completeness of SES and RUC data

There were no missing SES and RUC measures in both databases at the practice level for the UK. Across the combined CPRD GOLD and CPRD Aurum databases, 13 724 412 patients were currently registered in England and, among these, 12 805 555 patients (93%) had at least one patient-level measure (ie, IMD composite and domains, Carstairs, Townsend and/or RUC). A small percentage (6.7%, n=918 857) of currently registered acceptable patients had no patient-level measures available across the combined CPRD GOLD and CPRD Aurum data.

### Agreement between CPRD patient-level and practice-level SES and RUC data in England

**Figure 2:**
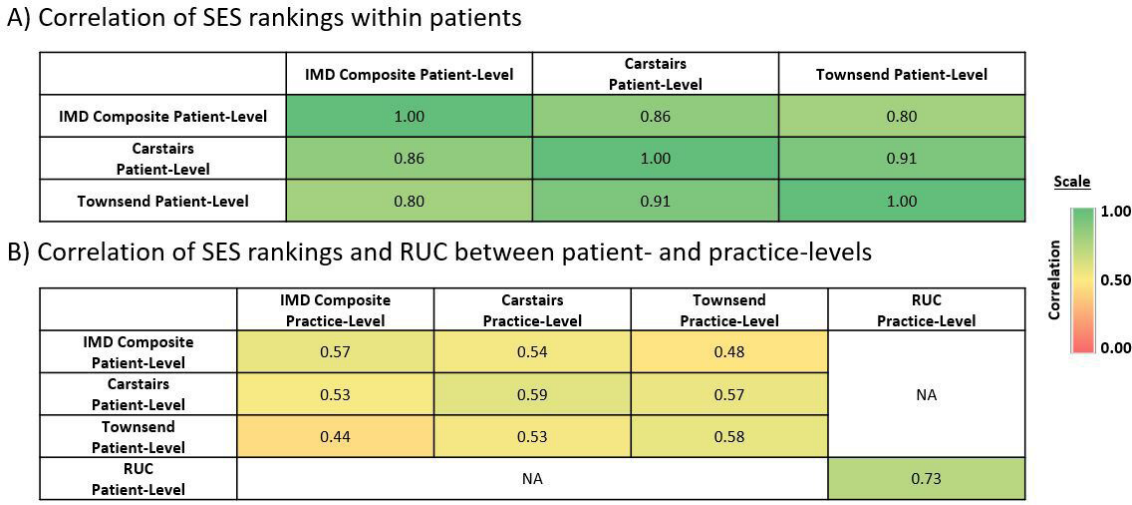
Correlation between socioeconomic status (SES) rankings (A) within patients and (B) between patients and their practice. Correlations between the Index of Multiple Deprivation (IMD) Composite, Carstairs Index and Townsend Deprivation Index both within patients and between patients and their practice. Correlation for rural–urban classification (RUC) between patients and their practice. Correlations in the Clinical Practice Research Datalink (CPRD) acceptable patient population from England only (CPRD GOLD and CPRD Aurum, combined) in May 2021. NA, not applicable.

Among the 12 805 555 acceptable, currently registered patients in England with a patient-level measure in the combined databases, high agreement (r≥0.80) between patient-level quintile rankings by each SES metric (IMD composite, Townsend and Carstairs) were observed (figure 2A). Agreement between patient-level IMD domains was highest between the income and employment domains (r=0.91). High agreement was also seen between the income and education domains; the income and health domains; the employment and education domains; the health and employment domains; and the housing and living environment domains (r≥0.75; online supplemental figure 2).

**Figure 3:**
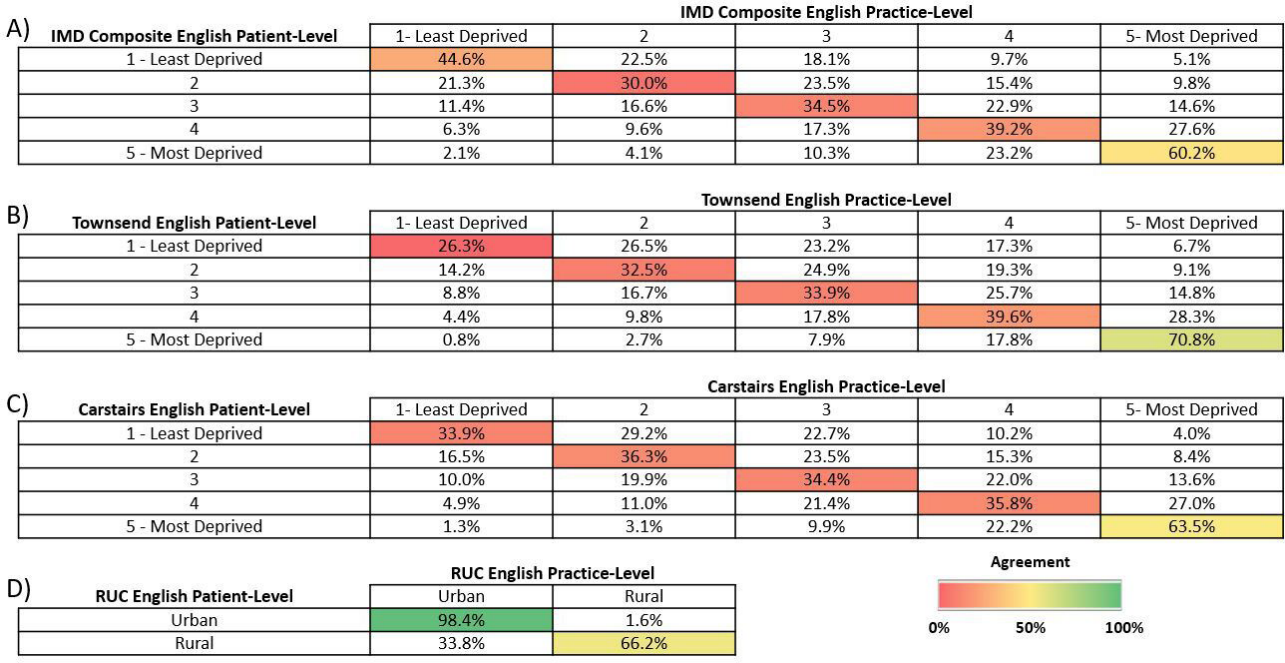
Proportion of patients with the same or different deprivation level as their practice per the (A) Index of Multiple Deprivation (IMD) Composite, (B) Carstairs Index and (C) Townsend Deprivation Index, or (D) rural–urban classification (RUC) in the Clinical Practice Research Datalink (CPRD) acceptable patient population from England only (CPRD GOLD and CPRD Aurum, combined) in May 2021. Colouring shows the level of agreement (%) between patient-level rank and practice-level rank.

Overall, fewer than 45% of patients in England were assigned to the same SES rank quintile (based on their residence postcode) as their GP practice. This was consistently observed among the different SES measures. The highest levels of agreement between patient and practice ranks were observed between the most deprived ranks (~60%–70%) (figure 3). Patient-level and practice-level SES rankings were only moderately correlated, both within and between SES metrics (r=0.44–0.59; figure 2B). Patient-level and practice-level rankings were only slightly correlated between the different IMD domains (r<0.50) and moderately correlated within the same IMD domain (r=0.51–0.81; online supplemental figure 3).

RUC measures were the most highly correlated measure between patient- and practice-levels (r=0.73; figure 2B) with 98.4% of patients classified as urban were registered at a practice also classified as urban (figure 3).

### Representativeness of the CPRD-linked SES and RUC data

**Figure 4:**
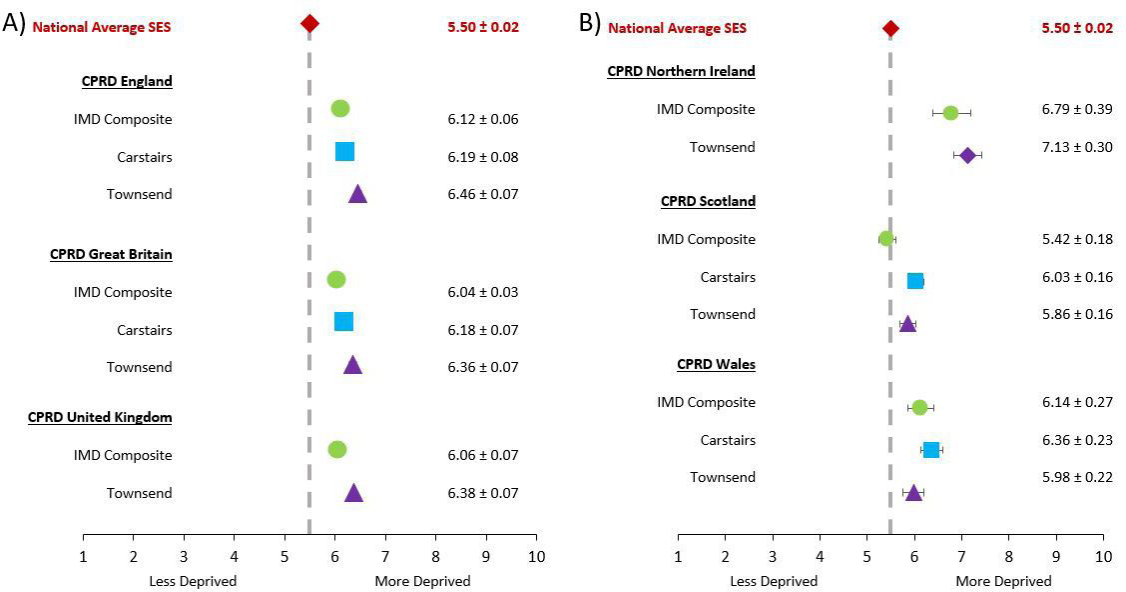
Representativeness of practice-level socioeconomic status (SES) measures—Index of Multiple Deprivation (IMD) Composite, Carstairs Index and Townsend Deprivation Index—in the Clinical Practice Research Datalink (CPRD) (CPRD GOLD and CPRD Aurum, combined) in terms of average decile of deprivation ±SD for all currently contributing practices in (A) England, Great Britain and the UK and (B) Northern Ireland, Scotland, and Wales in May 2021. The Carstairs Index is not available for Northern Ireland or the UK.

The average decile deprivation ranks of CPRD practices in all geographies were more deprived compared with the average decile deprivation ranks of the general populations in each geography (figure 4A, B).

**Figure 5:**
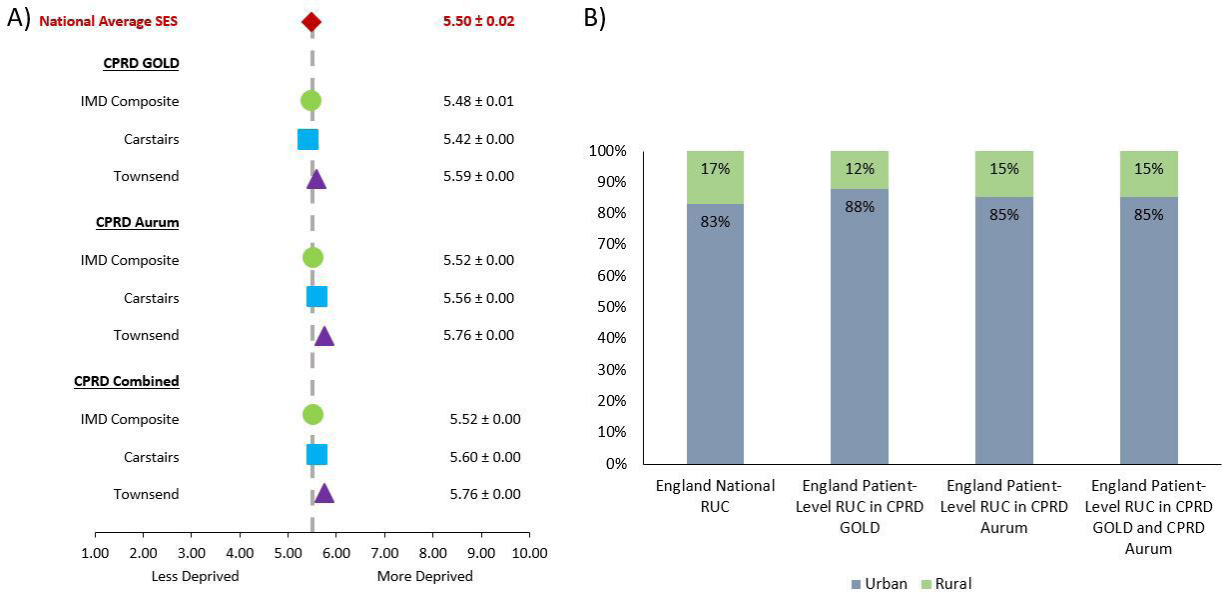
(A) Representativeness of patient-level socioeconomic status (SES) measures—Indices of Multiple Deprivation (IMD) Composite, Carstairs Index and Townsend Deprivation Index—in the Clinical Practice Research Datalink (CPRD); CPRD GOLD and CPRD Aurum, individually and combined) in terms of average decile of deprivation ±SD for all acceptable, currently registered patients in England in May 2021. (B) Representativeness of patient-level rural–urban classification (RUC) in CPRD (CPRD GOLD and CPRD Aurum, individually and combined) in terms of proportion (%) rural and urban for all acceptable, currently registered patients in England in May 2021 compared with the national measure.

The average decile deprivation ranks of CPRD patients in England were comparable to the defined average ±SD decile deprivation rank of England’s general population (5.50±0.02) for all SES measures and in each database (figure 5A).

There was a higher percentage of urban CPRD practices in England, Northern Ireland and Wales as compared with the total percentage of urban practices in these nations; whereas, CPRD practices in Scotland were more rural compared with the total percentage for Scottish practices (online supplemental figure 4). In England, CPRD patients lived in predominantly urban areas, broadly similar to the overall English profile (85% in urban areas CPRD vs 83% nationally) (figure 5B).

## Discussion

This study provides further insights into the completeness and representativeness of CPRD-linked area-level SES and RUC data. Overall, SES data for patients and practices linked to CPRD GOLD and CPRD Aurum data was broadly similar to the UK population at the time of analysis.

### Completeness and correlation of CPRD-linked SES data

Area-level SES and RUC measures could be assigned to 100% of the practice postcodes for GPs registered with CPRD GOLD and CPRD Aurum and area-level SES and RUC measures could be assigned to approximately 93% of patients postcodes in England across the CPRD GOLD and CPRD Aurum databases combined. The availability of patient-level measures depends on the following factors: practice consent to participate in the CPRD patient-level linkage scheme; patients must not have a record indicating dissent for transmission of their personal confidential data to NHSD; and a full valid postcode of residence recorded for the patient in primary care.^8 10^ Missing data at the patient level is a result of these additional criteria not being met.

There was high correlation between the patient-level SES data between the IMD composite, Townsend, and Carstairs measures as well as between some patient-level IMD domains. As these measures are highly correlated and the provision of multiple measures increases the risk of reidentification of patients and practices, most studies would be served by including only one patient-level and/or one practice-level area-level measure to assess deprivation.^23^ When selecting a SES measure for a study, researchers should consider variables used to derive SES ranks for example, variables related to material deprivation and external validity, allowing results to be most comparable with other published work.

### Poor agreement between SES data at patient level and practice level

The overall agreement and correlation between practice-level and patient-level SES was lower compared with the within patient-level measures. Studies in the past have used CPRD practice-level SES data as a proxy for missing patient-level data^24 25^; however, this may introduce bias. Since January 2015, GP practices in England have been free to register new patients who live outside their practice boundary area.^26^ Therefore, the lower agreement may be due to patients registering at a practice near their place of work or education, rather than near their home. Another factor can be due to a GP having their main site postcode held by CPRD but have associated branch practices contribute data to CPRD using the same main site postcode. Where these branched practices are in different geographical areas, the SES of these branch practice patients may not correlate with the SES of the main site.

Historic patient postcodes are not maintained in GP records or held by CPRD, and therefore, all linkages to small areas conducted by NHSD using the NHSPD are based on the currently recorded postcode for a patient. It cannot be known if the postcode in the GP records reflects the patient’s current or historical residing address. Thus, some assumptions must be made when utilising patient area-level SES measures: (1) the postcode in their GP record is current and may not reflect historical exposure and (2) patient experiences the same deprivation as their small area average.

Researchers should consider whether a patient area-level SES measure is a suitable proxy for an individual-level SES measure in their studies.^27^ Researchers are asked to consider how these assumptions may affect their results and to note these in the methodology and/or limitations of any resulting output.

When using practice-level data to estimate a patient’s SES, one must assume that the patient experiences the same level of deprivation as the small area in which their GP’s main site is located. Studies where patient area-level SES is a main exposure may wish to limit their study population to patients in England with patient-level SES measures, using both databases combined to minimise missingness. Studies wishing to use patient area-level SES as a descriptor, stratifying variable and/or covariate may judge whether the practice area-level measure is a sufficient proxy for their purposes; however, it is recommended that this be noted in the methodology and/or limitations of any resulting output. To further investigate this, researchers may wish to conduct sensitivity analysis using patient-level and practice-level SES to see the impact on the resulting outputs.

In contrast to the SES measures, the correlation and agreement between the practice-level and patient-level RUC were high. In most situations, practice-level RUC could be used as a proxy for patient RUC, as judged by the investigators. Again, it is recommended that the use of practice-level RUC as a proxy for patient-level RUC is noted in the methodology and/or limitations of any resulting output.

### CPRD practices are in more deprived areas of the UK

This study confirms that the average CPRD-linked patient-level SES in CPRD GOLD and CPRD Aurum databases, individually and combined, are similar to that of the general population in England.

At practice level, the SES data linked via CPRD GOLD and CPRD Aurum databases, combined, are more deprived than their respective geographies: England, Northern Ireland, Scotland and Wales. According to Wolf *et al*,^6^ the CPRD Aurum patient population was slightly less deprived in comparison to the English population. Since that publication in September 2018, CPRD has recruited more practices from more deprived areas in England into the CPRD Aurum database, with the average IMD composite of these recruited practices being 6.51 (SD ±0.12). This has led to slightly more GP practices in deprived areas in CPRD Aurum compared with the national distribution of GPs. The completeness and the representativeness of CPRD data may continue to change over time as CPRD recruits new practices. Thus, it will be important for future public health research to repeat these analyses and compare findings.

### Limitations

In this descriptive study, IMD and RUC were aggregated to the GB and UK geographies; however, it is important for researchers to note that the classifications for these measures for each nation are ranked independently within each nation and are not directly comparable between nations (figure 1). IMD and RUC should not be used for between nation comparisons in non-descriptive analyses without adjustment.^28^

While CPRD does maintain complete IMD and RUC data for all practices, due to technical processing some database builds may output with a small number of practices missing IMD or RUC data. This study used the latest CPRD-linked SES and RUC measures SES measures have been persistent over time and the updated metrics are highly correlated to historic metrics.^12^ For example, the Spearman’s rank correlation coefficient for quantiles, is 0.97, between the IMD composite classifications English IMD 2010 and 2015 and 0.98 between Scottish IMD 2009 and 2012 and 0.93 between NI 2010 and 2017 and 0.97 between Welsh IMD 2011 and 2014 (data not shown).^23^

As discussed above, sometimes patients will not be linked to SES or RUC data for any number of reasons, resulting in missing data. Researchers may employ several methodologies to address such missing data, including complete-case analysis, multiple imputations, maximum likelihood-based formulations, full Bayesian models and weighting methods.^29–32^

This study investigated the completeness and representativeness of the IMD composite measure. For further understanding of the representation of this deprivation measure, researchers can investigate this at the more granular level of IMD domains. The IMD composite measure is derived from of a number of indicators covering different aspects (‘domains’) of material deprivation. CPRD can provide quantiles of a specific IMD domain at patient and practice level if a specific domain would be a more meaningful metric of deprivation for a particular study.

## Conclusions

Overall, this study confirms that the completeness of the CPRD-linked area-level SES is high and there is a broad representativeness of the patient populations in CPRD in terms of SES and RUC compared with the general population of the UK. The study provides advice for researchers using deprivation measures in combination with CPRD, supporting them to make appropriate choices on use of small area data for public health research and benefit.

## Data Availability

Data may be obtained from a third party and are not publicly available. This study is based in part on data from CPRD obtained under licence from the UK Medicines and Healthcare products Regulatory Agency (MHRA). The data are provided by patients and collected by the National Health Service (NHS) as part of their care and support. The interpretation and conclusions contained in this study are those of the authors alone. The data that support the findings of this study are available from CPRD, but restrictions apply to the availability of these data, which were used under license for the current study, and so are not publicly available. Requests to access CPRD data are reviewed via the CPRD Research Data Governance (RDG) Process to ensure that the proposed research is of benefit to patients and public health. More information is available on the CPRD website: https://www.cprd.com/safeguarding-patient-data. This study used data from the May 2021 builds of CPRD GOLD and CPRD Aurum with linked SES and RUC data from linkage set 22. On reasonable application to the CPRD RDG, researchers may use this information to assemble the data used in this study. For further information, please contact the study authors in the first instance.

## References

[1] Stringhini S , Carmeli C , Jokela M , et al . Socioeconomic status and the 25 × 25 risk factors as determinants of premature mortality: a multicohort study and meta-analysis of 1·7 million men and women. The Lancet 2017;389:1229–37. 10.1016/S0140-6736(16)32380-7 PMC5368415 28159391

[2] Stafford M , Marmot M . Neighbourhood deprivation and health: does it affect us all equally? Int J Epidemiol 2003;32:357–66. 10.1093/ije/dyg084 12777420

[3] Mouratidis K . Neighborhood characteristics, neighborhood satisfaction, and well-being: the links with neighborhood deprivation. Land use policy 2020;99:104886. 10.1016/j.landusepol.2020.104886

[4] Clinical Practice Research Datalink . CPRD gold may 2021 (version 2021.05.001), 2021. Available: https://www.cprd.com/cprd-gold-may-2021-dataset

[5] Clinical Practice Research Datalink . CPRD Aurum May 2021 dataset (Version 2021.05.001) [Data set], 2021. Available: https://www.cprd.com/cprd-aurum-may-2021-dataset

[6] Wolf A , Dedman D , Campbell J , et al . Data resource profile: clinical practice research Datalink (CPRD) aurum. Int J Epidemiol 2019;48:1740–1740g. 10.1093/ije/dyz034 30859197 PMC6929522

[7] Clinical Practice Research Datalink . CPRD aurum small area data (practice) January 2022 (version 2022.01.001), 2022. Available: https://www.cprd.com/cprd-aurum-small-area-data-practice-january-2022

[8] Clinical Practice Research Datalink . CPRD aurum small area data (patient) January 2022 (version 2022.01.001), 2022. Available: https://www.cprd.com/cprd-aurum-small-area-data-patient-january-2022

[9] Clinical Practice Research Datalink . CPRD gold small area data (practice) January 2022 (version 2022.01.001), 2022. Available: https://www.cprd.com/cprd-gold-small-area-data-practice-january-2022

[10] Clinical Practice Research Datalink . CPRD gold small area data (patient) January 2022 (version 2022.01.001), 2022. Available: https://www.cprd.com/cprd-gold-small-area-data-patient-january-2022

[11] Office for National Statistics . Census geography, 2022. Available: https://www.ons.gov.uk/methodology/geography/ukgeographies/censusgeography

[12] Ministry of Housing Communities & Local Government, Office for National Statistics . English indices of deprivation 2019, 2019. Available: https://www.gov.uk/government/statistics/english-indices-of-deprivation-2019

[13] Scottish Government, Housing and Social Justice Directorate . Scottish index of multiple deprivation 2020, 2020. Available: https://www.gov.scot/collections/scottish-index-of-multiple-deprivation-2020/

[14] Northern Ireland Statistics and Research Agency (NISRA) Gníomhaireacht Thuaisceart Éireann um Staitisticí agus Taighde . Northern Ireland multiple deprivation measure 2017 (NIMDM2017), 2017. Available: https://www.nisra.gov.uk/statistics/deprivation/northern-ireland-multiple-deprivation-measure-2017-nimdm2017 [Accessed 29 Mar 2022].

[15] Stats Cymru StatsWales, Llywodraeth Cymru Welsh Government . Welsh index of multiple deprivation, 2019. Available: https://gov.wales/welsh-index-multiple-deprivation-full-index-update-ranks-2019 [Accessed 29 Mar 2022].

[16] Office for National Statistics, National Records of Scotland, Northern Ireland Statistics and Research Agency, UK Data Service . 2011 UK Townsend deprivation scores, 2017. Available: https://statistics.ukdataservice.ac.uk/dataset/2011-uk-townsend-deprivation-scores

[17] Wheeler B . Carstairs index 2011 for Lower-layer super output areas UK Data Service ReShare; 2014. https://reshare.ukdataservice.ac.uk/851497/

[18] Northern Ireland Statistics and Research Agency (NISRA) Gníomhaireacht Thuaisceart Éireann um Staitisticí agus Taighde . Urban - Rural Classification, 2016. Available: https://www.nisra.gov.uk/support/geography/urban-rural-classification [Accessed 29 Mar 2022].

[19] Rural and Environment Science and Analytical Services Division, Scottish Government . Scottish government urban rural classification 2016, 2018. Available: https://www.gov.scot/publications/scottish-government-urban-rural-classification-2016/pages/1/ [Accessed 29 Mar 2022].

[20] Office for National Statistics . Rural urban classification (2011) of lower layer super output areas in England and Wales, 2018. Available: https://data.gov.uk/dataset/b1165cea-2655-4cf7-bf22-dfbd3cdeb242/rural-urban-classification-2011-of-lower-layer-super-output-areas-in-england-and-wales [Accessed 29 Mar 2022].

[21] Smith L , Downing A , Norman P , et al . Influence of deprivation and rurality on patient-reported outcomes of men living with and beyond prostate cancer diagnosis in the UK: a population-based study. Cancer Epidemiol 2020;69:101830. 10.1016/j.canep.2020.101830 33002843

[22] Office for National Statistics, NHS Digital . Nhs Postcode directory UK full (November 2017), 2017. Available: https://geoportal.statistics.gov.uk/datasets/5b0817e0bc5a4aaf817050b91e2bc385/about [Accessed 29 Mar 2022].

[23] Clinical Practice Research Datalink . CPRD linked data, 2022. Available: https://www.cprd.com/linked-data

[24] Rafiq M , Hayward A , Warren-Gash C , et al . Socioeconomic deprivation and regional variation in Hodgkin's lymphoma incidence in the UK: a population-based cohort study of 10 million individuals. BMJ Open 2019;9:e029228. 10.1136/bmjopen-2019-029228 PMC6756616 31542744

[25] Hire AJ , Ashcroft DM , Springate DA , et al . ADHD in the United Kingdom: regional and socioeconomic variations in incidence rates amongst children and adolescents (2004-2013). J Atten Disord 2018;22:134–42. 10.1177/1087054715613441 26604267

[26] National Health Service . Registering with a GP surgery outside the area you live, 2018. Available: https://www.nhs.uk/nhs-services/gps/registering-with-a-gp-outside-your-area/

[27] Boyle PJ , Gatrell AC , Duke-Williams O . Do area-level population change, deprivation and variations in deprivation affect individual-level self-reported limiting long-term illness? Soc Sci Med 2001;53:795–9. 10.1016/S0277-9536(00)00373-7 11511054

[28] Abel GA , Barclay ME , Payne RA . Adjusted indices of multiple deprivation to enable comparisons within and between constituent countries of the UK including an illustration using mortality rates. BMJ Open 2016;6:e012750. 10.1136/bmjopen-2016-012750 PMC5128942 27852716

[29] Bhaskaran K , Forbes HJ , Douglas I , et al . Representativeness and optimal use of body mass index (BMI) in the UK clinical practice research Datalink (CPRD). BMJ Open 2013;3:e003389. 10.1136/bmjopen-2013-003389 PMC3773634 24038008

[30] Martín-Merino E , Calderón-Larrañaga A , Hawley S , et al . The impact of different strategies to handle missing data on both precision and bias in a drug safety study: a multidatabase multinational population-based cohort study. Clin Epidemiol 2018;10:643–54. 10.2147/CLEP.S154914 29892204 PMC5993167

[31] Karahalios A , Baglietto L , Carlin JB , et al . A review of the reporting and handling of missing data in cohort studies with repeated assessment of exposure measures. BMC Med Res Methodol 2012;12:96. 10.1186/1471-2288-12-96 22784200 PMC3464662

[32] Secrest MH , Platt RW , Reynier P , et al . Multiple imputation for systematically missing confounders within a distributed data drug safety network: a simulation study and real-world example. Pharmacoepidemiol Drug Saf 2020;29 Suppl 1:35–44. 10.1002/pds.4876 31486165

