# Supplementary material for: Completeness and representativeness of small area socioeconomic data linked with the UK Clinical Practice Research Datalink (CPRD)
Source: J Epidemiol Community Health. 2022 Jul 28;76(10):880–6. doi: 10.1136/jech-2022-219200 (PMC9484378; doi:10.1136/jech-2022-219200)
Supplement: Supplementary data [file jech-2022-219200supp001.pdf]

Supplementary Material

Completeness and representativeness of small area socioeconomic data linked with the UK Clinical Practice Research Datalink (CPRD)

Authors: Preveina Mahadevan[1*] (ORCID- ID: 0000-0001-5856-3365), Mia Harley[1], Stuart Fordyce[1], Susan Hodgson[1] (0000-0001-8519-8586), Rebecca E Ghosh[1] (ORCID ID: 0000-0001-6009-3040), Puja Myles[1] (ORCID-ID: 0000-0002-8976-890X), Helen P Booth[1] (ORCID ID: 0000-0002-5866-864X), Eleanor L Axson[1] (ORCID ID: 0000-0001-9494-2389)

[1]Clinical Practice Research Datalink (CPRD), Medicines and Healthcare products Regulatory Agency (MHRA), 10 South Colonnade, Canary Wharf, London, E14 4PU, United Kingdom

*Corresponding Author: preveina.mahadevan@mhra.gov.uk

Supplementary Figure 1

Definition of Acceptable Patients for Research in the Clinical Practice Research Datalink (CPRD)

Patients are labelled as 'acceptable' for use in research by a process that identifies and excludes patients with non-continuous follow up or patients with poor data recording that raises suspicion as to the validity of the that patients record. Patient data is checked, for the following issues:

- An empty or invalid first registration date
- An empty or invalid current registration date
- Absence of a record for a year of birth
- A first registration date prior to their birth year
- A current registration date prior to their birth year
- A transferred-out reason with no transferred-out date
- A transferred-out date with no transferred-out reason
- A transferred-out date prior to their first registration date
- A transferred-out date prior to their current registration date
- A current registration date prior to their first registration date
- A gender other than Female/Male/Indeterminate
- An age of greater than 115 at end of follow up
- Recorded health care episodes in years prior to birth year
- All recorded health care episodes have empty or invalid event dates
- Registration status of temporary patients

If any of these conditions are true, then the patient is labelled unacceptable and is not recommended for use in research.

Over 98% of permanent registrations are deemed to have 'acceptable' (or research quality) data based on CPRD metrics.

BMJ Publishing Group Limited (BMJ) disclaims all liability and responsibility arising from any reliance placed on this supplemental material which has been supplied by the author(s)

Correlation (0.00 0.50 1.00)	Patient-Level IMD Domain								
Patient-Level IMD Domain	Income	Employment	Education	Health	Crime	Access to Services*	Living Environment**	Housing**	Outdoor Environment**
Income	1.00	0.91	0.76	0.75	0.64	0.10	0.26	0.15	0.29
Employment	0.91	1.00	0.77	0.80	0.59	-0.02	0.18	0.14	0.17
Education	0.76	0.77	1.00	0.69	0.49	-0.03	0.09	0.10	0.07
Health	0.75	0.80	0.69	1.00	0.57	-0.13	0.22	0.22	0.12
Crime	0.64	0.59	0.49	0.57	1.00	0.07	0.33	0.19	0.45
Access to Services*	0.10	-0.02	-0.03	-0.13	0.07	1.00	0.21	0.01	0.31
Living Environment**	0.26	0.18	0.09	0.22	0.33	0.21	1.00	0.82	0.51
Housing**	0.15	0.14	0.10	0.22	0.19	0.01	0.82	1.00	0.11
Outdoor Environment**	0.29	0.17	0.07	0.12	0.45	0.31	0.51	0.11	1.00

Supplementary Figure 2. Correlation between English Index of Multiple Deprivation (IMD) domain rankings within patients. Correlations in the Clinical Practice Research Datalink (CPRD) acceptable patient population from England only (CPRD GOLD and CPRD Aurum, combined) in May 2021. *The name of this domain has been changed by CPRD to standardise naming across measures; the 'access' domain here is also known as the 'barriers to housing and services' domain. **The 'living environment' domain is made up of two sub-domains for 'indoor' and 'outdoor' local environments, here these sub-domains are titled 'housing' and 'outdoor environment', respectively, and are offered by CPRD as domains.

Correlation (0.00 – 0.50 – 1.00)	Patient-Level IMD Domain								
**Practice-Level IMD Domain**	**Income**	**Employment**	**Education**	**Health**	**Crime**	**Access to Services***	**Living Environment****	**Housing****	**Outdoor Environment****
**Income**	0.51	0.50	0.41	0.46	0.38	0.04	0.18	0.11	0.18
**Employment**	0.47	0.52	0.43	0.49	0.32	-0.06	0.11	0.11	0.04
**Education**	0.38	0.43	0.51	0.44	0.27	-0.10	0.06	0.10	-0.01
**Health**	0.42	0.48	0.42	0.64	0.36	-0.18	0.16	0.20	0.03
**Crime**	0.41	0.39	0.30	0.42	0.53	0.06	0.21	0.10	0.32
**Access to Services***	0.17	0.04	-0.01	-0.06	0.19	0.53	0.26	0.03	0.45
**Living Environment****	0.24	0.20	0.08	0.26	0.26	0.08	0.51	0.39	0.36
**Housing****	0.12	0.14	0.09	0.25	0.10	-0.11	0.33	0.42	-0.01
**Outdoor Environment****	0.28	0.16	0.06	0.12	0.38	0.29	0.41	0.07	0.81

Supplementary Figure 3. Correlation between English Index of Multiple Deprivation (IMD) domain rankings between patients and their practice. Correlations in the Clinical Practice Research Datalink (CPRD) acceptable patient population from England only (CPRD GOLD and CPRD Aurum, combined) in May 2021. *The name of this domain has been changed by CPRD to standardise naming across measures; the ‘access’ domain here is also known as the ‘barriers to housing and services’ domain. **The ‘living environment’ domain is made up of two sub-domains for ‘indoor’ and ‘outdoor’ local environments, here these sub-domains are titled ‘housing’ and ‘outdoor environment’, respectively, and are offered by CPRD as domains.

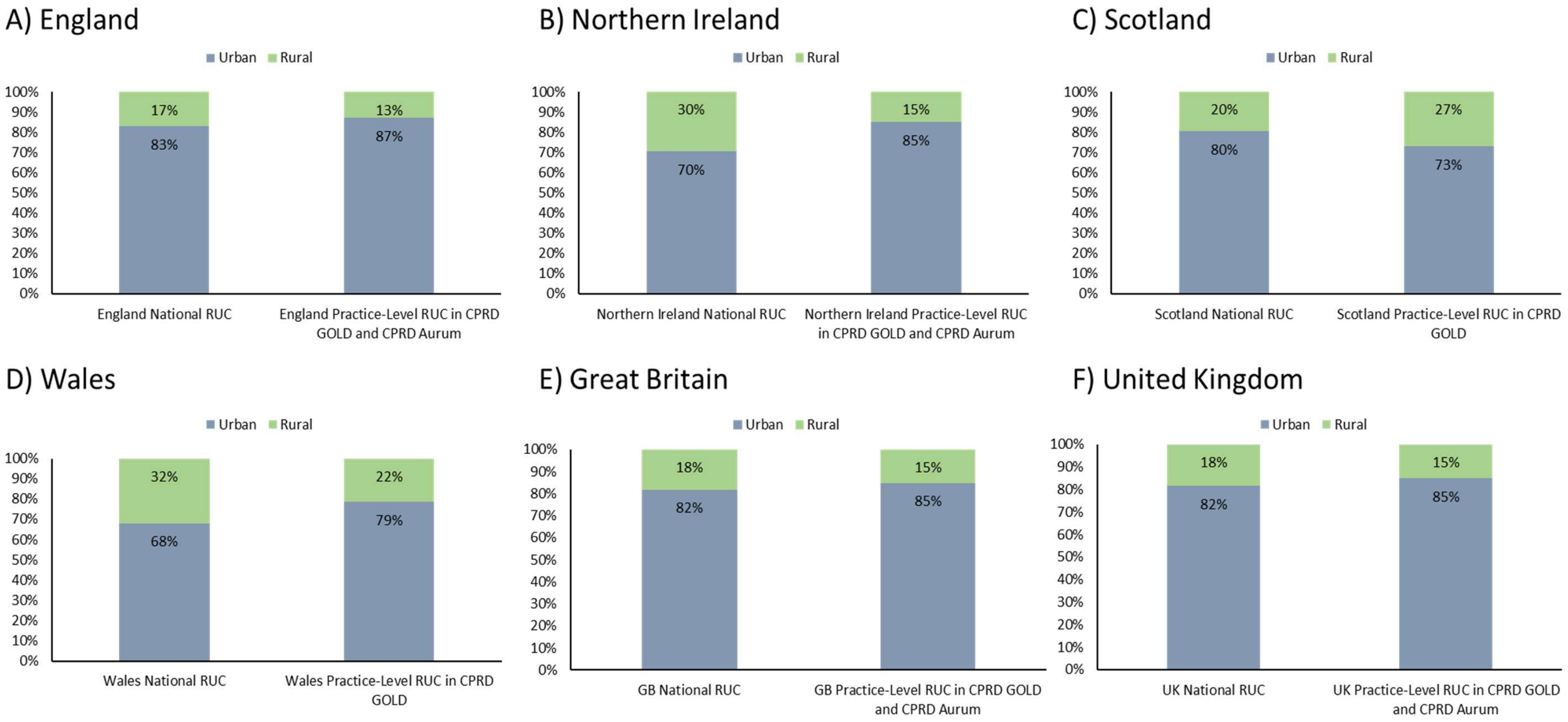

Supplementary Figure 4. Representativeness of Rural-Urban Classification (RUC) in the Clinical Practice Research Datalink (CPRD; CPRD GOLD and CPRD Aurum, combined) in terms of proportion (%) rural and urban for all currently contributing practices in **A)** England, **B)** Northern Ireland, **C)** Scotland (CPRD GOLD only), **D)** Wales (CPRD GOLD only), **E)** Great Britain (GB), and **F)** the United Kingdom (UK) in May 2021 compared to the national measures.
